# Cobalamin C deficiency: a rare but treatable genetic cause of pulmonary hypertension

**DOI:** 10.1186/s13023-025-03903-0

**Published:** 2025-07-10

**Authors:** Julien Grynblat, Florence Coulet, David Montani

**Affiliations:** 1 INSERM UMR_S 999 “Pulmonary Hypertension: Pathophysiology and Novel Therapies”, Marie Lannelongue Hospital and Bicêtre Hospital, Le Plessis-Robinson, France; 2 Department of Respiratory and Intensive Care Medicine, AP-HP, Pulmonary Hypertension National Referral Center, Bicêtre Hospital, Le Kremlin-Bicêtre, France; 3 School of Medicine, University of Paris-Saclay, Le Kremlin- Bicêtre, France; 4 AP-HP, M3C-Necker, Hôpital Necker-Enfants Malades, Université de Paris Cité, Cardiologie Congénitale et Pédiatrique, Paris, France; 5 Département de génétique médicale, Sorbonne Université, Assistance Publique- Hôpitaux de Paris, Hôpital Pitié-Salpêtrière, Paris, France

**Keywords:** Cobalamin C, Genetics, Pulmonary hypertension, Pulmonary veno-occlusive disease

Pulmonary hypertension (PH) is a hemodynamic condition defined by an increase in pulmonary arterial pressure (PAP). The diagnosis relies on invasive assessment by right heart catheterization, with a mean PAP (mPAP) > 20 mmHg [1, 2]. PH encompasses several etiologies, including pulmonary arterial hypertension (PAH), a rare form of PH characterized by progressive remodeling and obstruction of the pulmonary microcirculation. Other causes of PH include PH due to left heart disease, PH associated with lung diseases and/or hypoxia, PH associated with pulmonary artery obstructions, and PH with unclear and/or multifactorial mechanisms [1, 2]. Among the forms of PAH, heritable PAH is of particular interest. A genetic cause can be identified in approximately 70–90% of familial PAH cases and in 12–20% of sporadic cases, with heterozygous germline pathogenic variants in the *BMPR2* (*Bone morphogenetic protein receptor type 2)* gene accounting by far the most common genetic predisposition [3–5]. These heritable forms often exhibit pronounced pulmonary arterial remodeling at a younger age, and a severe, progressive course, without curative treatment options [6].

Cobalamin C (CblC) deficiency, the most commonly encountered inborn error of cobalamin metabolism, impairs the conversion of dietary cobalamin into methylcobalamin and adenosylcobalamin, due to *MMACHC* gene homozygous or compound heterozygous variants [7–9]. This impaired enzymatic activity leads to homocysteine and methylmalonic acid accumulation, with methionine synthesis. Clinical manifestations are diverse, ranging from early-onset disease (symptoms beginning before 1 year of age) to late-onset presentations. General features include cytopenias, renal failure, dysmorphism, failure to thrive, and feeding difficulties. In addition, neurological manifestations are quite frequent and include hypotonia, developmental delay, and even psychiatric symptoms among adults.

In an issue of the *Orphanet Journal of Rare Diseases*, Ding et al. are to be commended for presenting the largest cohort to date (26 patients) with CblC deficiency and PH [10]. All patients underwent genetic testing for CblC deficiency, confirming loss-of-function variants in *MMACHC with inactivation of the two alleles*. However, a major limitation lies in the diagnostic criteria for PH, which were based solely on echocardiographic estimates (systolic PAP > 40 mmHg), without confirmatory invasive hemodynamic data. As previously established, right heart catheterization (RHC) remains essential for confirming the diagnosis of PH, determining its underlying mechanism, and assessing its severity [1, 2, 11]. PH (mPAP > 20 mmHg) is classified as precapillary PH when pulmonary vascular resistance (PVR) is > 2 Woods unit (WU) and pulmonary arterial wedge pressure (PAWP) is ≤ 15 mmHg and postcapillary PH when PVR is < 2 WU and PAWP > 15 mmHg. In children, a PVR index > 3 WU/m² defines precapillary PH. Notably, RHC is an invasive procedure and is associated with in pediatric population with non-negligible procedural risk: major adverse events in 3.5–6.2% and mortality between 0.2 and 1.4%, even in expert centers [12, 13]. Risk factors include general anesthesia, age under 2 years, organ dysfunction, and suprasystemic PH. Given the young age of the patients reported by Ding et al., it is understandable that RHCs were not performed; however, this limits the generalizability of their findings, particularly in patients with congenital heart disease (CHD).

Nonetheless, this study estimated the prevalence of PH in 1.1% (26/2352 patients) of individuals with CblC deficiency [10]. Patients’ characteristics included a male predominance (sex ratio M/F 1.4), and a median age at PH diagnosis of 3.25 years (range from 1 month to 13.5 years). Remarkably, PH was the first clinical manifestation of CblC deficiency in 61% of cases. Most patients also presented with extracardiac and extrapulmonary manifestations, including developmental delay, seizures, intellectual disability, anemia, thrombocytopenia, and renal involvement (e.g., proteinuria and/or renal thrombotic microangiopathy). CHD was also common, with atrial/ventricular septal defects, and pulmonary artery stenosis noted. Interestingly, several case reports describe adult patients with PH and CblC deficiency, typically presenting with varying degrees of mental retardation, renal insufficiency and thrombotic microangiopathy [14–20]. These observations suggest a broad age spectrum and support considering metabolic aetiologies in both pediatric and adult-onset PH.

Genetic advances have clarified the molecular landscape of PAH over the last two decades. Twelve genes are now recognized as causal for PAH, half of which belong to the transforming growth factor β (TGF-β) superfamily, and particularly *BMPR2* [3, 21]. Most forms are autosomal dominant with incomplete penetrance, except for pulmonary veno-occlusive disease (PVOD), which is inherited in an autosomal recessive pattern with near-complete penetrance [22–25]. Beyond the TGF-β pathway, other genes such as *TBX4* (*T-box 4*),*SOX17 (SRY-related HMG box 17*), and *KDR (kinase insert domain receptor)* involved in pulmonary and cardiac development, have also been implicated [26–28]. The study by Ding et al. underlies the importance of extra pulmonary and/or extra cardiac features as potential diagnostic clues to uncover rare, potentially reversible causes of PH. The relative low penetrance of PH among patients with CblC deficiency suggests that additional factors, such as environmental triggers, individual susceptibility, or genetic modifiers may be necessary for disease development. This hypothesis aligns with findings in *BMPR2* mutation carriers, where the annual incidence of PAH is 2.3%, and most carriers do not develop PAH during their lifetime [4]. Notably, although Ding et al. identified the c.80 A > G variant in approximately 70% of patients, its specific association with PH remains unconfirmed, as it was not overrepresented in previously published CblC-related PH cases [10]. Furthermore, the authors did not discuss the recessive aspect of CblC deficiency, and most of the patients with the c.80 A > G variant did not develop PH.

This article, in addition to previous reports, also raises classification issues. Indeed, PH associated with CblC could theoretically fall into multiple groups: heritable PAH, PAH associated with CHD for a subset of patients, but also PH due to metabolic disorders (group 5 PH). Additionally, some cases suggest overlap with PVOD, a rare form of PAH, based on clinical and imaging features [14–16]. Due to the rarity of cases, the presence of associated comorbidities, and the potential for reversibility, patients with CblC-related PH are generally not considered for lung transplantation. As a result, histopathological data are insufficient to precisely characterize the pulmonary vascular lesions in this condition. Pulmonary vascular remodelling in PAH is characterized by an abnormal proliferation of smooth pulmonary artery cells, and endothelial cell dysfunction, which leads to an occlusion of the small pulmonary vessels [29]. In CblC deficiency, the mechanisms underlying PH development among patients are unknown, nevertheless endothelial cell dysfunction associated with fibrosis as found in the kidneys of patients with CblC deficiency may lead to severe respiratory consequences such as PH.

Prompt recognition of this rare association is essential—particularly in the presence of developmental delay, renal dysfunction, or CHD, but also in cases of apparently isolated paediatric PH. Biochemical screening is straightforward, and *MMACHC* mutations can be confirmed via genetic testing. Early diagnosis leads to specific treatment, which may alter the disease course significantly. In Ding et al. study, treatment included hydroxycobalamin, L-carnitine, betaine, and folinic acid [10]. Twelve patients also received PAH-approved drugs. After a follow-up ranging from 6 months to 7 years, two patients died due to multiple organ failure and severe heart failure; however, the remaining cases were alive and all but one patient showed normalized PAP on echocardiography [10].

In conclusion, PH associated with CblC deficiency is a treatable and potentially reversible cause of PH. It should be systematically considered in children and young adults with PH, especially when associated with developmental delay, CHD, and renal insufficiency. In suspected cases. RHC remains indispensable, whenever feasible, in suspected cases to confirm the diagnosis and guide therapy. Of note, PH is often the first symptom that could support the inclusion of metabolic screening (homocysteine, methylmalonic acid) in the diagnostic workup for idiopathic or atypical PH. When CblC deficiency is confirmed, prompt metabolic treatment should be initiated, with PAH-approved drugs in non-responders and severe cases. Finally, the inclusion of *MMACHC* in next-generation sequencing (NGS) panels for PAH warrants discussion, especially in pediatric cases and in the presence of syndromic features.
